# Metformin—A Type 2 Diabetes Mellitus Drug—And Ovarian Cancer: Anticancer Mechanisms and Therapeutic Implications

**DOI:** 10.3390/biom16030413

**Published:** 2026-03-11

**Authors:** Emma Sielski, Al-Noumani Shuhd, Ella Bower, Kate Cunningham, Grace Beidel, Alissa Luchianova, Maria Cecilia Courreges, Fabian Benencia

**Affiliations:** 1 Department of Biomedical Sciences, College of Osteopathic Medicine, Academic Research Center, Ohio University, Athens, OH 45701, USA; es099121@ohio.edu (E.S.); gb311522@ohio.edu (G.B.); al604022@ohio.edu (A.L.); 2 Biomedical Engineering Program, Department of Chemical and Biomolecular Engineering, Russ College of Engineering and Technology, Ohio University, Athens, OH 45701, USA; sa489020@ohio.edu; 3 Clinical and Translational Research Unit, Research and Grants, HCOM, Ohio University, Athens, OH 45701, USA; 4 Molecular and Cell Biology Program, Ohio University, Athens, OH 45701, USA; 5 Diabetes Institute, Ohio University, Athens, OH 45701, USA; 6 Institute for Molecular Medicine and Aging, Ohio University, Athens, OH 45701, USA

**Keywords:** metformin, type-2 diabetes mellitus, high-grade ovarian cancer, tumor microenvironment, chemotherapy

## Abstract

Ovarian cancer is a devastating disease that is often diagnosed in the late stages. The typical therapeutic approach includes surgery plus cytotoxic drugs such as carboplatin and paclitaxel. In recent years, the advent of poly ADP-ribose polymerase (PARP) inhibitors such as olaparib has offered additional treatment opportunities for patients with BRCA mutations or homologous recombination deficiencies. Nevertheless, resistance to therapy usually occurs, leading to poor overall survival. Therefore, novel treatments are needed for this disease. One of the obstacles to successful treatment is the highly immunosuppressive nature of the ovarian cancer microenvironment. Recent strategies for the treatment of ovarian cancer and other types of cancer involve targeting the metabolism of cancer cells and other cells of the tumor microenvironment. One drug that has been investigated both in preclinical studies and clinical trials as an antitumor agent is metformin. This drug, typically used for the treatment of type-2 diabetes for its capability to lower blood glucose, can directly affect cancer cell growth and survival by activating the AMPK (adenosine monophosphate-activated protein kinase) pathway. Furthermore, it can affect the phenotype of other cells of the tumor microenvironment such as macrophages and T cells. In this review, we summarize the main characteristics of ovarian cancer and describe preclinical studies and clinical trials involving metformin as a therapeutic agent for this disease.

## 1. Introduction

According to the World Cancer Research Fund, in 2022 there were about 320,000 new cases of ovarian cancer, with the USA, India and China harboring the highest number of them. The American Cancer Society estimates that in 2025 about 20,000 women received a diagnosis of ovarian cancer in the USA, and that around 13,000 will die from this disease. Epithelial ovarian cancer (EOC) accounts for approximately 90% of ovarian cancer cases and is characterized by presentation at late stages, resulting in poor survival outcomes, with about 40% and 20% of women on average surviving less than 5 years after diagnosis (stage III and IV, respectively) [1]. Therefore, there is a need for novel therapeutic approaches for this disease. Herewith, we review the use of metformin as a single drug or as part of combinatorial therapies for ovarian cancer treatment.

## 2. Epithelial Ovarian Cancer Generalities and Treatment

The vast majority of ovarian cancers are of epithelial origin. Non-epithelial ovarian cancer tumors include germline tumors, sex cord tumors or small cell tumors, among others [1]. High-Grade Serous Ovarian Cancer (HGSOC) is the most common histological subtype of epithelial ovarian cancer. Other epithelial ovarian cancers are categorized into low-grade serous, endometrioid, clear cell, and mucinous [2,3]. HGSOC is often diagnosed at later stages due to its asymptomatic nature and it frequently recurs after therapy, with a median overall survival of less than five years. HGSOC usually affects patients older than 60 years of age and is typically detected in stage III–IV. These are poorly differentiated tumors that rarely express estrogen receptors [1].

As reviewed by Bhattacharya et al. [4], HGSOC is a challenging cancer for researchers and doctors due to its complicated genetics and high resistance to chemotherapy. It is considered that this disease can originate in the ovarian surface epithelium or develop from serous tubal intraepithelial carcinoma (STIC) lesions located in the fimbria of the fallopian tube, with cancer stem cells being found at both locations [5,6,7,8]. Indeed, experimental models of ovarian cancer can originate in the ovaries or tubes [9,10,11]. HGSOC typically presents as bilateral solid tumors that metastasize throughout the abdominal cavity, leading to the development of malignant ascites and microtumors in the omentum and other abdominal organs. During peritoneal metastasis, which historically has been considered the predominant route of dissemination, cancer cells released from the primary tumor site circulate in the peritoneal fluid and implant in and invade the mesothelial layer of abdominal organs and the omentum. Patients also generate malignant ascites that help spread the tumor throughout the peritoneal cavity. Ovarian cancer cells in the ascites are resistant to anoikis, a type of cell death induced by absence of contact with the surrounding extracellular matrix [8]. One of the reasons for this resistance is the constitutive activation of integrins by the cancer cells [12]. As discussed below, tumor cells in ascites have a different metabolic status compared to those in the primary tumor or when attached to the omentum or other organs. In some patients, the mesothelial layer of the omentum is intact over omental metastasis, highlighting an alternative route of metastasis in which the omental microtumors originate from underneath the peritoneal surface, where they arrive through lymphatic or vascular routes (hematogenous or lymphatic metastasis). It is also hypothesized that ovarian cancer metastasis can happen through nerve dissemination [8,13].

The standard-of-care first-line treatment for advanced ovarian cancer is surgery to remove the tumor followed by six cycles of a combination of platinum- (e.g., carboplatin) and taxane-based (e.g., paclitaxel) chemotherapy [14]. A significant factor in progression-free survival is the efficacy of the surgical procedure (debulking surgery), which depends, among other factors, on successful access to metastases and achieving optimal debulking with no residual disease [15,16]. In most patients, recurrence is observed within three years. The resistance is typically classified as platinum-sensitive if it occurs after six months of treatment completion or platinum-resistant if it happens within six months of treatment.

Tumor heterogeneity is a major problem in the treatment of HGSOC. As previously reviewed by Santoro et al. [17], HGSOC is characterized by high chromosomal instability mostly associated with mutations in the TP53 gene, which account for more than 90% of all somatic mutations in HGSOC [18]. Furthermore, BRCA1 and BRCA2 mutations are reported in about 40% of patients with HGSOC [19,20]. These mutations range from somatic mutations to germline mutations to epigenetic modifications (i.e., methylation of the BRCA1 promoter), alterations in BRCA gene expression, or impairment of pathways associated with DNA repair. Indeed, approximately half of HGSOC patients present defects in the homologous recombination repair (HRR) mechanism [17]. Failure of the HRR mechanism induces accumulation of genomic aberrations and is associated with cancer development and progression. In addition to BRCA gene mutations, defects in the expression and function of RAD51C, RAD51D, and BRIP1 genes are also considered risk factors for ovarian cancer because they interact with BRCA proteins to repair DNA double-strand breaks [21,22].

As a result of the disruption of the normal HRR mechanism, DNA double-strand breaks accumulate in the affected cells, creating a target that can be exploited with specific therapies. Poly(ADP-ribose) polymerase (PARP) is involved in repairing single-strand DNA breaks. PARP inhibitors (PARPi) block this function, thereby inducing the accumulation of single-strand breaks in the DNA. Single-strand breaks are converted to double-strand breaks during DNA replication, which are then repaired by HRR mechanisms [17]. If the cell has a defective HRR mechanism, it is unable to repair them, which leads to genomic instability and cell death. As described above, until the advent of PARPi, the standard of care for first-line treatment of advanced ovarian cancer consisted of cytoreductive surgery followed by chemotherapy. Although an effective initial response is observed with this treatment, the disease recurs in most patients. PARPi treatment improves progression-free survival for patients with HR-deficient tumors [17,23]. Unfortunately, patients also develop resistance to PARPi, and therefore novel treatments for ovarian cancer are needed [24,25,26].

HGSOC is also characterized by harboring genomic deletions or amplifications. These alterations appear to be more frequent than mutations, and amplification of MAPK15, MYC or CCNE1 genes was detected in about 30% of ovarian cancer samples [1,27].

It is noteworthy that although HGSOC has high genetic heterogeneity [3], the level of tumor mutational burden (TMB) is low [28]. TMB is defined as the total number of somatic non-synonymous mutations present within the cancer genome [29]. A low TMB will determine a low likelihood of neoantigen appearance, thereby presenting limited targets for the adaptive antitumor immune response or specific antigen-driven immunotherapies such as immune checkpoint inhibitor therapy or dendritic cell vaccination. A low TMB might contribute to the low efficacy of immune checkpoint blockade inhibitor therapy in ovarian cancer [30]. Indeed, tumor mutational burden is evaluated as a possible biomarker for predicting responses to immunotherapy.

Taking all of this into account, new strategies are needed for the treatment of ovarian cancer. One such strategy is to target metabolic pathways in cancer cells, in particular those associated with glucose metabolism.

## 3. Glucose Metabolism in Ovarian Cancer Cells

Ovarian cancer cells reprogram their glucose metabolism to survive hypoxia, enhance their proliferative capacity and escape the antitumor immune response. Like many other tumor cells, ovarian cancer cells favor aerobic glycolysis (Warburg effect) even in the presence of oxygen [31]. This allows cells to produce ATP faster (although less efficiently) than by the oxidative phosphorylation pathway supporting high levels of proliferation. It also helps cells adapt to the hypoxic environment of growing tumors, and it creates metabolites such as lactic acid that induce an acidic environment that modifies the ECM by activating matrix metalloproteases and also induces immunosuppression by skewing macrophages towards an M2 phenotype or directly affecting T cell and NK cell metabolism [32]. In particular, HGSOC cells overexpress glucose transporter type 1 (GLUT1), which allows capturing glucose from the environment in a more efficient manner [33,34,35].

Interestingly, there are some differences in the metabolic behavior of ovarian cancer cells in solid tumors and ascites. In solid tumors, the cells are subjected to more hypoxic environments than ascites, so in the latter, cancer cells have a more flexible metabolism with an increase in oxidative phosphorylation mechanisms, thereby generating lower amounts of lactic acid [36]. Not all ascites cells are oxidative phosphorylation-dependent; certain ascites-derived tumor subpopulations show upregulation of glycolysis (e.g., elevation of PDK4) and form glycolytic tumorspheres, which highlights the metabolic heterogeneity among ovarian cancer patient tumors [37].

Taking this into consideration, different therapeutic strategies have been investigated targeting glucose metabolism in ovarian cancer. Among others, studies have focused on blocking glycolytic flux by inhibiting PFKFB3, which is an enzyme that drives fructose-2,6-bisphosphate and high glycolytic throughput and is elevated in ovarian cancer [38]; neutralizing lactate signaling by inhibiting MCT1 or LDHA [38,39]; and impairing metabolic pathways that support glycolysis such as the HIF-1 signaling pathway [40] or the PI3K/AKT/mTOR axis with a metabolic stressor such as metformin [41].

## 4. Mechanism of Action of Metformin

Metformin is a synthetic biguanide administered orally for the purpose of achieving extended glycemic control for those living with Type 2 Diabetes Mellitus (T2DM). Chemically speaking, at physiological pH, metformin is an organic cation in the body; thus, its uptake into target tissues is dependent on the expression of organic cation transporters, plasma membrane monoamine transporters and multidrug and toxin extrusion proteins [42]. While its biological targets and mechanisms of action have been long debated, recent findings suggest that metformin targets the gastrointestinal tract, gut microbiota, and tissue resident immune cells, in addition to what many believe to be the traditional target of metformin, the liver [43,44,45,46].

Different mechanisms of action have been proposed for metformin’s inhibition of hepatic glucose production in the liver. The first is that metformin decreases gluconeogenesis by inhibiting mitochondrial respiratory chain complex 1. This determines a drop in ATP levels and a rise in AMP levels, which activates AMP kinase (AMPK). The AMPK pathway is both an energy-conservation and ATP-generating pathway that restores and maintains homeostasis. It primarily attains this function by regulating intracellular ratios of AMP:ATP and ADP:ATP. The enzyme phosphorylates and inhibits key transcription factors such as hepatocyte nuclear factor 4 (HNF4) and CREB-regulated transcription coactivator 2 (CRTC2) that promote the expression of gluconeogenic enzymes like phosphoenolpyruvate carboxykinase (PEPCK) and glucose 6-phosphatase (G6Pase) [47,48].

Metformin also acts through AMPK-independent pathways by inhibiting enzymes like fructose-1,6-bisphosphatase and mitochondrial glycerophosphate dehydrogenase [49]. Another suggested mechanism is that this biguanide can inhibit the activity of mitochondrial glycerol-3-phosphate dehydrogenase [50]. It has also been proposed that metformin can interact and bind to presenilin enhancer 2 (PEN2) on the surface of lysosomes. PEN2 interacts with a complex of membrane proteins that regulate the AMPK pathway. Therefore, binding of PEN2 to metformin activates the AMPK pathway, leading to the secretion of GLP1 and ultimately a reduction in blood glucose levels [42,51,52].

An increasing number of studies have examined the relationship between metformin and long non-coding RNA activity, specifically in pancreatic cells and hepatocytes. In this context, an association between metformin and upregulation of DNA methylation was found. Specifically, metformin increased the activity of DNA methyltransferase 1 (DNMT1), subsequently increasing methylation. This was not true for other cell types, indicating that the effects of metformin on lncRNA are dependent on cell identity [53].

In addition to reducing hyperglycemia, metformin is also known to reduce both microvascular and macrovascular complications, such as neuropathy and cardiovascular disease, respectively, associated with T2DM [54,55,56]. Preclinical studies have also demonstrated that metformin can have beneficial therapeutic effects in polycystic ovary syndrome, non-alcoholic fatty liver disease, liver cirrhosis, as well as numerous different types of cancer [54,57,58,59].

**Figure 1:**
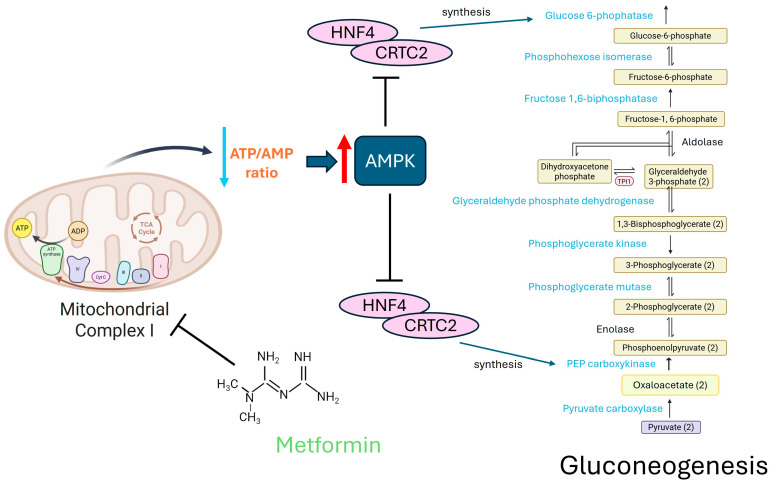
Effect of metformin on liver gluconeogenesis (AMPK-dependent). The figure summarizes the effect of metformin on liver gluconeogenesis mediated by AMPK activation. Red upward arrow: increase; light blue downward arrow: decrease. ADP: adenosine diphosphate, AMP: adenosine monophosphate, AMPK: AMP-activated protein kinase, ATP: adenosine triphosphate, CRTC2: CREB-regulated transcription coactivator 2, HNF4: hepatocyte nuclear factor 4, TCA: tricarboxylic acid cycle. Created with BioRender.com.

A summary of metformin mechanisms of action on liver gluconeogenesis (AMPK-dependent) is presented in Figure 1.

## 5. Metformin as an Anti-Tumor Agent

It has been reported that metformin can directly affect tumor cell growth and survival. This effect can be attributed to a reduction in oxidative phosphorylation, which will affect cells with high mitochondrial dependence (e.g., certain ovarian, prostate, and breast cancer cells). This can also lead to an increase in toxic reactive oxygen species production due to blockade of the mitochondrial complex I [60]. Additionally, activation of AMPK by metformin will in turn inhibit mTORC1, thus suppressing anabolic growth signals and protein synthesis, therefore slowing proliferation and inducing a senescent-like stage [61,62]. In different cancer cell lines, metformin is able to inhibit lipogenesis and nucleotide biosynthesis and trigger autophagy [63,64,65]. In addition, some reports indicate that metformin also has a deleterious effect on cancer stem cells [66].

## 6. Metformin and Ovarian Cancer Cells

In most studies investigating the effect of metformin on cancer cells, it has been shown that metformin interferes with the AMPK/mTOR axis, indicating that the drug affects protein synthesis and cell metabolism [67,68]. This axis is under the control of LKB1, a serine-threonine kinase with tumor suppressor activities [67]. As described above, metformin—which can enter cancer cells through organic cation transporters (OCTs)—inhibits the mitochondrial complex I, decreasing the ATP/AMP ratio in the cells. This in turn activates the AMPK pathway in normal and cancer cells, which determines the inhibition of the mTOR pathway [67].

In numerous in vitro studies, it has been demonstrated that metformin has antiproliferative and pro-apoptotic activity in different ovarian cancer cell lines (SKOV3, OVCAR3, OVCAR4, A2780 or HO8910) or primary cultures [69,70,71,72,73,74,75,76]. Some of these studies indicate that treatment with the drug induced ovarian cancer cell cycle arrest in the G0/G1 and S phases [69,70,73,77]. In addition, some of these studies also report that apoptotic key molecules were affected by metformin. Upregulation of apoptotic markers such as Bax and Bad, downregulation of Bcl-2 and Bcl-xL expression, and activation of caspases 3/7 were observed in treated cells [69,75]. Furthermore, a study using SKOV3 and A2780 cells in vitro and in vivo showed that, in addition to inducing ovarian cancer cell death, the drug activated the AMPK pathway in these cells [78]. The effect of metformin on AMPK pathway activation in ovarian cancer cells was reported in several in vitro studies and in mouse models of ovarian cancer [77,79,80,81,82,83,84]. Interestingly, the effect of metformin on ovarian cancer cells was counteracted by treatment with an AMPK inhibitor, further supporting a role of metformin in activating the AMPK signaling pathway in ovarian cancer cells [72].

Individual studies proposed additional mechanisms involved in the effect of metformin on ovarian cancer cells. One study reported that under low glucose conditions, metformin induced cell death via activation of apoptosis signal-regulating kinase 1 (ASK1), which in turn triggered JNK, leading to mitochondrial and endoplasmic reticulum stress [85]. This induced sustained ER stress and cell apoptosis in cancer cells but not in normal cells. Another study, using SKOV3 cells, showed that metformin was able to reduce the expression of pro-survival signals by decreasing mRNA and protein levels of Axl and Tyro3, two members of the TAM family of receptor tyrosine kinases (RTKs), and the anti-apoptotic protein XIAP (a caspase inhibitor) [86]. Furthermore, in another study, increased expression of autophagy marker LC3alpha was also observed in SKOV3 ovarian cancer cells treated with metformin [74]. Another study showed that metformin downregulates TRIM37 in ovarian cancer cells. This molecule, which promotes ubiquitination of TRAF2, is overexpressed in ovarian cancer cells and is linked to proliferation and invasion [87]. Metformin was also able to inhibit mesothelin in ovarian cancer cells in a different study, leading to a reduction in the IL-6/STAT3 signaling pathway [88]. Furthermore, in another study, increased expression of MUL1 E3 ligase, a molecule that targets AKT for proteasomal degradation, was reported in ovarian cancer cells after treatment with the drug [89]. In A2780 and SKOV3 cells, metformin decreased the nerve growth factor-induced transcriptional activity of MYC and beta-catenin/T-cell factor/lymphoid enhancer-binding factor (TCF-Lef), as well as the expression of c-MYC, survivin and VEGF [90].

Finally, in vitro and in vivo (xenograft) studies using ovarian cancer cells A2780 and SKOV3 showed that under low glucose conditions, metformin was able to induce apoptosis and ferroptosis in these cells by targeting NDUFB4, and thus inhibiting mitochondrial complex I, increasing reactive oxygen species production and lipid peroxidation [91]. Interestingly, AMPK can exert anti-proliferative activity by impairing lipid biosynthesis via inhibition of acetyl co-carboxylase, an enzyme of the fatty acid synthesis pathway [92]. A limitation of some of the studies showing mechanisms of action of metformin beyond AMPK signaling modulation is that the observed changes in cellular metabolism remain largely associative. Further studies focusing on experiments such as gene overexpression, knockdown/knockout and rescue assays, and inhibition with pharmacological inhibitors, as described in AMPK signaling studies, would provide stronger support for the proposed mechanisms [72]. In addition, some of the studies are based on a limited set of experimental models; for example, limited variety of in vitro cellular systems. Therefore, more investigation needs to be conducted to determine if the proposed mechanisms of action can be replicated in models with different genetic backgrounds, baseline pathway activation, or cellular states.

In summary, although several studies indicate the capability of metformin to decrease proliferation and induce apoptosis in ovarian cancer cells, and activate AMPK signaling, there is no consensus regarding further mechanisms affected by the drug in these cells.

**Figure 2:**
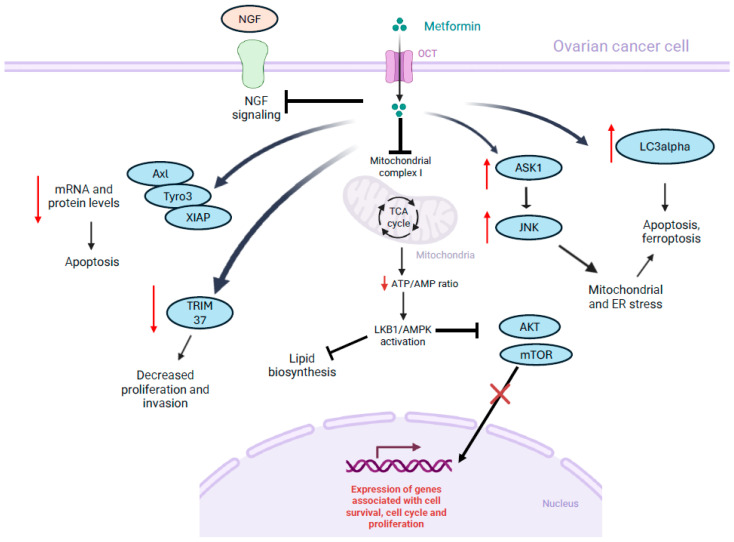
Proposed effects of metformin on ovarian cancer cells. The figure summarizes reported targets of metformin in ovarian cancer cells. NGF: nerve growth factor, OCT: organic cation transporters, TCA: tricarboxylic acid cycle. Red downward arrows: downregulation; Red upside arrows: upregulation. Created with BioRender.com.

Figure 2 summarizes some of the proposed mechanisms of action of metformin in ovarian cancer cells described in this section.

## 7. Metformin and Ovarian Cancer Stem Cells

Studies in mouse ovarian cancer cells showed that tumor-initiating cells possess reduced basal glucose and fatty-acid oxidation metabolism but increased lactate secretion, which is consistent with a glycolytic shift. They present metabolic plasticity by increasing glycolysis when ATP synthase is inhibited and raise maximal oxygen consumption rate after uncoupling, indicating capacity to engage both glycolysis and oxidative phosphorylation when needed. Tumor-initiating cells survive better under nutrient-limiting conditions and are more sensitive to metformin when compared with normal ovarian surface epithelial cells [93]. Studies using human ovarian cancer cells (including SKOV3) showed that metformin reduced the fraction of cells expressing stem cell markers (CD44/CD117/CD133 as determined by flow cytometry), downregulated EMT transcription factors Snail2 and Twist, limited their capacity to generate spheres, and reduced migration in vitro [94].

In xenograft models, metformin reduced tumor-initiatng cell frequency and synergized with cisplatin to slow tumor growth [95]. Importantly, a Phase II clinical trial was established to evaluate the use of metformin to target cancer stem cells in ovarian cancer. The study was a single-arm Phase II in non-diabetic patients with advanced epithelial ovarian cancer (stage IIC–IV). Metformin was given with standard chemotherapy, and the primary translational endpoints included change in cancer stem cell markers (ALDH^+^ CD133^+^) and ex vivo chemosensitivity. The study showed a 2.4-fold reduction in tumor ALDH^+^ CD133^+^ cell fraction after metformin treatment, improved ex vivo cisplatin sensitivity of tumor cells, and epigenetic reprogramming of carcinoma-associated mesenchymal stromal cells toward less chemoresistant phenotypes [96].

## 8. Effects of Combinatorial Treatments Using Metformin and Cytotoxic Agents on Ovarian Cancer Cells

Metformin has been studied in combination with anti-tumor agents in ovarian cancer. For example, in OVCAR-3 and SKOV3 cells it has been shown that combining simvastatin (statin) with metformin was effective in inducing a stronger growth inhibition, less migration, and higher apoptosis than both drugs alone [97]. Combining metformin and simvastatin acted on PIK3R1, affecting the mTOR pathway. PIK3R1 mediates the activation of the PI3K/AKT signaling pathway, and thus its inhibition decreases tumor cell metabolism, affecting cell proliferation [97].

Metformin was also able to increase the sensitivity of ovarian cancer cells to chemotherapeutic drugs. For example, metformin increased the sensitivity of human ovarian cancer cell lines to cisplatin, paclitaxel, or methotrexate [98,99]. In a study using the human ovarian HO-8910 cancer cell line, it was shown that the combination of metformin plus cisplatin was able to increase apoptosis of cancer cells more efficiently than both drugs alone, downregulating the expression of p-ERK1/2, VEGF, VEGFR2 and Bcl-2, while increasing the expression of Bax and caspase-3 [100].

Furthermore, metformin was able to increase sensitivity to chemotherapy in mouse models of ovarian cancer. In this study, a syngeneic orthotopic model of ovarian cancer was used, and metformin was given in the water together with paclitaxel, which was administered intraperitoneally. It was shown that treatment with paclitaxel plus metformin was more effective than using both drugs alone [101]. It was also shown that metformin increased the sensitivity of ovarian cancer cells to the apoptotic effects of the PARP inhibitor Olaparib [102].

On the other hand, metformin might antagonize the effect of other anti-tumor agents. It has been shown that metformin decreased the anti-neoplastic efficacy of the IGF-1 receptor inhibitor linsitinib in OVCAR3 cells [103].

## 9. Effects of Combinatorial Treatments Using Metformin and Metabolic Agents on Ovarian Cancer Cells

A study combining metformin with dichloroacetate (DCA), a compound that targets mitochondria and increases the activity of pyruvate dehydrogenase kinase, thus reversing mitochondrial dysfunction, was performed in SKOV3 and OVCAR3 ovarian cancer cells. The study showed that the combination was able to suppress proliferation and migration of these cancer cells [104]. The rationale for this combination is that targeting two energy pathways simultaneously—the glycolytic pathway (DCA) and mitochondrial oxidative phosphorylation (metformin)—could result in a stronger effect. The results of the study showed that DCA sensitized the anti-tumor function of metformin, thus providing a rationale for the use of both agents in combinatorial therapies [104].

A study combining metformin with 2-Deoxyglucose (2DG) showed that they inhibited growth, migration, invasion and induced cell cycle arrest of ovarian cancer cells in vitro (SKOV3 and Hey) [105]. This might be because, while metformin alone could increase glycolysis to compensate for mitochondrial inhibition, combining metformin with 2-DG prevents this escape mechanism. Indeed, it was shown that while metformin and 2-DG alone decreased intracellular ATP concentration by about 60% in SKOV3 and 40% in Hey cells, the combination of the drugs diminished ATP concentration by about 90% in these cell lines. Similar results were obtained in two other studies in which 2-DG increased the antitumor effect of metformin in different ovarian cancer cell lines [80,106]. This suggests that it might be relevant to further investigate the use of this combinatorial approach in other cell lines and in mouse models of ovarian cancer.

Studies were performed to compare the efficacy of metformin with another biguanide, phenformin, a lipophilic biguanide, on human ovarian cancer cells (SKOV3, Hey, IGROV-1), patient-derived primary cell lines and in an orthotopic xenograft model of ovarian cancer. It was shown that phenformin had significantly higher potency than metformin in growth inhibition at both low and high dosages. Its increased efficacy was attributed to the drug’s ability to enter cells without relying on OCT1 unlike metformin [107]. On the other hand, phenformin has been retired from the market due to its association with fatal lactic acidosis, and there are no clinical trials listed involving phenformin for ovarian cancer.

Another metabolic drug that has been used in ovarian cancer studies is lonidamine, which inhibits mitochondrial-bound hexokinase II, therefore affecting glycolysis, and also induces mitochondrial dysfunction. There are currently no studies either comparing the efficacy of both drugs or using them in combination for ovarian cancer. Lonidamine has been shown to enhance the effect of cisplatin, carboplatin or Taxol in ovarian cancer cell lines and primary cultures of patient-derived ovarian cancer cells, and ovarian cancer xenografts (SKOV3, IGROV-1) in nude mice [108,109,110,111]. Phase I/II clinical trials with lonidamine in ovarian cancer showed that combination with carboplatin, cisplatin and lonidamine was tolerable for the treatment of advanced ovarian cancer (25 patients) and that it had an effect on reverting resistance to platinum (27 patients) [112,113]. There are currently no clinical trials for ovarian cancer involving lonidamine listed in ClinicalTrials.gov. It would be of interest to define the combinatorial capacity of metformin plus lonidamine in ovarian cancer.

## 10. The Tumor Microenvironment (TME) of Ovarian Cancer: Generalities and Targets for Metformin Action

The tumor microenvironment (TME) is composed of tumor cells, the extracellular matrix (ECM) and the stroma. In the case of ovarian cancer, tumor cells metastasize within the peritoneal cavity, and in addition to a solid tumor, malignant ascites is induced. The ascites environment differs from that of solid tumors [114]. It has been shown that ascites affects the integrity of the mesothelium and promotes the implantation of tumor spheroids in the mesothelium of the omentum, favoring metastasis [114]. The microenvironment of the solid tumor and of the ascites contributes to resistance to therapy in ovarian cancer.

HGSOC solid tumors are characterized by tumor islets surrounded by a distinctive stroma. The stroma contains cancer-associated fibroblasts (CAFs), endothelial cells and infiltrating immune cells [115]. Tumor-associated macrophages (TAMs) predominate among the immune infiltrate, but infiltration of other myeloid cells, such as myeloid-derived suppressor cells (MDSCs) and dendritic cells, is also observed. Different subsets of T cells and B lymphocytes are present in solid tumor and ascites, where they promote or oppose tumor development. The ECM of ovarian cancer is composed mainly of collagen, but other molecules, such as fibronectin, laminins and hyaluronan, are present [116]. The ECM of ovarian cancer not only provides mechanical support for the tumor and stromal cells but also acts as a reservoir of molecules that can affect tumor growth, such as vascular endothelial growth factor (VEGF), which can be released from the ECM by proteases produced by tumor or stromal cells.

The environment of ascites is characterized by fluid containing ECM components, growth factors and cytokines, and floating spheroids, which can harbor tumor cells, CAFs and TAMs. B cells are also found in the ascites, where they can have a positive or negative influence on tumor development [117,118].

## 11. The Extracellular Matrix (ECM) of Ovarian Cancer

Typical extracellular matrix components of ovarian cancer solid tumors are collagens (mainly types I, III, IV and VI), fibronectin 1, laminins, hyaluronan and thrombospondins. These molecules not only provide structural support to tumor or stromal cells, but also function as a reservoir of growth factors such as VEGF, epithelial growth factor or transforming growth factor beta (TGFβ), which can promote angiogenesis, proliferation and metastasis. ECM components are not distributed homogeneously. It was shown that while laminin γ1 was associated with tumor cells, collagen molecules generated fibrillar networks within the surrounding microenvironment [116]. A dense ECM will also provide a barrier for immune cell infiltration into tumors and can limit access of chemotherapeutic reagents to tumor cells. In the ascites, ECM components are associated with multicellular spheroids, facilitating cell interaction, providing tumor cell resistance to anoikis (apoptosis due to loss of contact to a surface), and also participating in adhesion to omentum and thus peritoneal dissemination [116].

Finally, ECM stiffness has a role in promoting epithelial-to-mesenchymal transformation (EMT). It has been shown that stiffness can activate mechanosensing pathways in tumor cells, which promote a more aggressive metastatic phenotype [119].

## 12. Cancer-Associated Fibroblasts (CAFs) in Ovarian Cancer

Among the stromal cells, CAFs play a relevant role in the biology of ovarian cancer [120]. CAFs usually derive from normal fibroblasts transformed under the influence of tumor factors [121]. Proteomics and single-cell transcriptomics have identified different CAF populations in HGSOC, which differ between solid tumor and ascites [115]. The multiple CAF populations identified at present in the ovarian cancer microenvironment, and also present in the microenvironment of other tumors, are myofibroblastic CAFs (myCAFs), inflammatory CAFs (iCAFs), antigen-presenting CAFs (apCAFs), mesothelial-like CAFs, adipocyte-derived CAFs and proliferative CAFs. MyCAFs are characterized by production of extracellular matrix components that help tumor development; iCAFs produce inflammatory factors, and apCAFs can present antigen to T cells via MHC-II. The latter two can promote or suppress tumor growth depending on the microenvironment. These three are the CAF populations present at the highest levels in ovarian cancer. While all three of these populations can be found in the stroma of solid tumors, myCAFs are rare in ascites, and apCAFs are found at higher levels in ascites than in solid tumors [115].

## 13. Endothelial Cells in Ovarian Cancer

As a tumor develops, it generates a vascular network through several mechanisms, including neoangiogenesis, vasculogenesis, or vasculogenic mimicry [122,123,124,125]. In ovarian cancer, as in other cancers, new vascular vessels are generated from existing vessels to nurture the tumors. This mostly happens through the budding of new blood vessels from existing microvascular beds [126]. This pathological process, named neoangiogenesis, is fueled by VEGF and other angiogenic factors, such as angiopoietin, produced by tumor cells or other cells of the microenvironment, such as macrophages [122,127]. This has provided a rationale for ovarian cancer therapies using bevacizumab, a humanized monoclonal antibody against VEGF [128,129]. It is noteworthy that in addition to its angiogenic role, VEGF is also directly involved in inducing tumor cell proliferation and promoting immune tolerance, with receptors expressed in cancer cells and immune cells, among others [128]. Clinical trials in ovarian cancer using bevacizumab have demonstrated benefits mainly when used in combination with chemotherapeutic drugs [128,130].

Tumor vascular vessels are different from normal endothelial vessels since they show leakiness and are tortuous [122]. The characteristics of these vessels also facilitate metastasis. In ovarian cancer, tumor endothelial cells can function as a barrier to immune cell response by inactivating T cells or preventing T cell adhesion and thus infiltration into tumors [131,132]. In addition, tumors can recruit bone marrow-derived precursor cells (endothelial and pericyte progenitor cells) from circulation to contribute to tumor vascularization, a process named vasculogenesis [122]. Finally, vasculogenic mimicry, a process in which tumor cells mimic endothelial functions, has also been reported in ovarian cancer [125,133,134,135,136].

Therapeutic strategies to normalize tumor vascular endothelium have been shown to promote an antitumor effect and allow for more efficient delivery of therapeutic drugs [126]. As reviewed by Yu et al. [126], anti-angiogenic therapy can induce vessel normalization, during which vessels undergo structural and functional changes, such as enhanced perfusion, improved pericyte coverage, and relieved hypoxia. These changes can alter the immunosuppressive nature of the TME. For example, normalized tumor vasculature diminishes tumor hypoxia, which can induce polarization of TAMs towards an M1-like phenotype, reduce recruitment of regulatory T cells (Treg) and MDSCs, and decrease hypoxia-induced PD-L1 expression [126,137]. In addition, vascular normalization has been shown to increase the efficacy of oncolytic virotherapy in a mouse model of ovarian cancer [138].

In addition, lymphatic vessels also play a role in ovarian cancer. A study assessing the effects of lympho-vascular space invasion on recurrence and survival in patients with primary epithelial ovarian cancer reported that this histologic feature is associated with more aggressive cancer behavior and is a predictor of survival in patients with early-stage ovarian cancer but not with advanced disease [139]. On the other hand, when lymphatic vessels are part of tertiary lymphoid structures containing T cells, B cells and follicular dendritic cells at tumor sites, this appears to correlate with a better response to immunotherapy, highlighting the complexity of the ovarian cancer microenvironment [140].

## 14. Cancer-Associated Adipocytes (CAAs) in Ovarian Cancer

Adipose cells are active components of the TME of ovarian cancer, where they can influence the biology of other cell types and promote tumor progression [141,142,143]. Adipose cells are themselves affected by the TME, undergoing morphological and functional changes (lipid depletion, fibroblastic phenotype, altered secretome) and becoming cancer-associated adipocytes (CAAs) [144]. In general, compared with typical mature adipocytes, CAAs exhibit an irregular shape, smaller volume, and small, dispersed lipid droplets. In epithelial ovarian cancer, adipocytes secrete adipokines (e.g., leptin, IL-6, IL-8, IL-33) and free fatty acids, and modulate epithelial-to-mesenchymal transformation (EMT), support cancer cell invasion, adhesion, angiogenesis and metastatic seeding in the omentum [145].

In ovarian cancer, CAAs might also contribute to chemotherapy resistance. For example, arachidonic acid secreted by adipocytes can activate the AKT signaling pathway in tumor cells and, therefore, impair cisplatin-induced apoptosis [144,146]. Furthermore, adipocyte products can decrease the efficacy of paclitaxel therapy in ovarian cancer [147]. In addition, adipocytes can modulate the metabolism of ovarian cancer cells. It was shown that co-culture of human adipocytes with ovarian cancer cells stabilized HIF1α expression in tumor cells, thereby increasing the glycolytic rate in these cells and rerouting glucose-derived carbons towards G3P and GPL synthesis [143]. It was shown that blocking this mechanism in tumor cells reduced metastasis in xenograft mouse models of ovarian cancer.

## 15. Tumor-Associated Macrophages (TAMs) in Ovarian Cancer

In HGSOC, TAMs are the most abundant immune population as determined by immunohistochemistry analysis of human tumor samples [148]. They derive either from resident macrophages (for example, peritoneal macrophages) or from monocytes recruited from the circulation [149]. In particular, solid tumor TAMs typically derive from monocytes, while TAMs in the ascites derive both from local peritoneal macrophages and recruited monocytes. M-CSF, CCL2, CCL5, VEGF and MIP-4 (CCL18) are some of the factors that can recruit monocytes to the ovarian cancer TME [149]. In the TME, these cells can adopt an M2-like phenotype under the influence of tumor factors such as TGF-β, IL-4, IL-10, or prostaglandin E2 (PGE2), among others [149]. Nevertheless, M1-like macrophages (CD68+, HLA-DR+) are also present in the ovarian TME, in particular in solid tumors [150,151,152]. Typically, M1-like macrophages are effective in tumor eradication, although they can be involved in promoting angiogenesis. M2-like TAMs can be found in hypoxic areas of solid tumors, but they predominate in the ascites, being relevant constituents of spheroids. M2-like TAMs (high CD163+ or CD206+) not only exert immunosuppressive effects but also facilitate angiogenesis, ECM remodeling, invasion, and metastasis [150,153,154,155]. It has been reported that in HGSOC patients, a higher ratio of M1 to M2 macrophages was associated with longer overall survival, progression-free survival and platinum-free intervals [150,156,157].

Single-cell RNA sequencing analysis identified four clusters of macrophages in HGSOC malignant ascites marked by CD14, AIF1, CSF1R and CD163 [115]. In another RNA sequencing study, it was described that, in this disease, an increase in M2 macrophages was associated with tumor progression and immune evasion [158]. A transcriptome analysis of ovarian cancer databases was used to divide samples into low-risk and high-risk based on a prognostic model. The study determined that the low-risk group had higher M1 macrophages and CD8^+^ T cell infiltration abundance, while the high-risk group had significantly higher abundances of M2 macrophage infiltration [159].

Overall, TAMs promote angiogenesis and tumor suppression in ovarian cancer and also participate in tumor metastasis. In particular, in the ascites, TAMs are key components of spheroids, helping tumor cells resist anoikis, promoting expression of peritoneal mesothelial cell adhesion molecules, and releasing growth factors and invasive proteases that contribute to peritoneal metastasis [160]. In addition to promoting tumor growth, TAMs contribute to chemotherapy resistance and thus have been considered a target for ovarian cancer therapies—in preclinical studies and clinical trials—aiming to eliminate or reprogram them [161,162,163,164]. Interventions include drugs that act as inhibitors of M2 macrophages or CCL2 production, molecules involved in recruitment of macrophages to the TME, antibodies that target CD47 (a “don’t eat me signal” expressed by tumor cells, which prevents phagocytosis by macrophages), or macrophage depletion with CART cells, among others [161,163].

## 16. Dendritic Cells (DCs) in Ovarian Cancer

Classical (or conventional) type 1 (cDC1) and type 2 (cDC2) dendritic cells have been identified in HGSOC, both in solid tumors and ascites [165,166,167]. cDC1s function as activators of CD8 T cells, while cDC2 enable CD4 T cell activation. DCs in circulation have been studied to determine the response to therapy. Indeed, it has been reported that circulating cDC1s are reduced in patients with ovarian cancer, while the activation of cDC2 is increased, and that the loss of the cDC1 subset might correlate with a poor prognosis [168]. Tumor-associated DCs might be in a state of immunological immaturity and might not be able to activate T cells adequately. In a preclinical model, it has been shown that tumor-associated DCs in ovarian cancer can contribute to tumor angiogenesis [169]. Inflammatory DCs, a population of DCs characterized by CD11c and HLA-DR expression but that do not align with the conventional DC phenotype by harboring gene signatures of monocyte-derived DCs, have been detected in ovarian cancer ascites, where they induce Th17 differentiation [170]. Interestingly, using mouse models of ovarian cancer, it has been shown that under appropriate conditions, tumor-associated DCs (that might already harbor tumor antigen) can be activated to induce antitumor immune responses [171].

## 17. Myeloid-Derived Suppressor Cells (MDSCs) in Ovarian Cancer

MDSCs are a heterogeneous group of immature myeloid cells that function as co-conspirators of tumor growth by dampening the immune response. They can suppress the activity of T cells and NK cells, thus enabling tumor cells to escape killing by these cytotoxic effectors [172]. MDSCs also promote cancer cell invasion, metastasis, and angiogenesis [173]. The presence of high levels of MDSCs in the tumor microenvironment is often associated with poor prognosis in ovarian cancer [174].

In ovarian cancer, MDSCs can be found within the solid tumor, the ascites and in circulation [174]. MDSCs can be broadly divided into Monocytic (M)-MDSCs and Polymorphonuclear (PMN)-MDSCs due to their phenotypic resemblance to those populations [172]. Although both populations have immunosuppressive effects, M-MDSCs are characterized by the production of immunosuppressive cytokines, while PMN-MDSCs produce high levels of reactive oxygen species, which can induce oxidative stress in immune cells [172]. Furthermore, it has been shown that they are able to promote epithelial ovarian cancer cell stemness, increasing the generation of spheroids and the expression of stem cell markers in tumor cells [175].

MDSCs are significantly increased in the peripheral blood and present in solid tumors or ascites in ovarian cancer patients [173]. They differentiate from bone marrow precursors and are attracted to tumors by locally produced chemokines such as CXCL8, CXCL12, and CCL5, among others [173]. It has been shown that an increased number of tumor-infiltrating MDSCs in HGSOC patients was associated with decreased survival and correlated with lower infiltration of CD8 T cells [173,174,176,177,178].

## 18. Tumor-Associated Neutrophils (TANs) in Ovarian Cancer

Neutrophils, innate immune cells strongly involved in first responses against infection, in particular of bacterial origin, also have a role in ovarian cancer development [179,180]. These innate immune cells are highly phagocytic, generate reactive oxygen species (ROS) when activated and can form neutrophil extracellular traps (NETs) when dying, a process called netosis.

Although it was considered that neutrophils were a homogeneous and terminally differentiated cell population of short-lived cells, a growing body of knowledge provides evidence that this is a heterogeneous population that differs in phenotype and function depending on the context. For example, some investigators consider that, in the TME, neutrophils can be divided into tumor-associated neutrophils-1 and -2 (N1 and N2 TANs), the former being antitumoral and the latter protumoral [181]. Neutrophils are a component of the ovarian cancer TME [179,180,182].

In ovarian cancer, multiple meta-analyses and cohort studies report that an elevated neutrophil-to-lymphocyte ratio (NLR) or high neutrophil infiltration correlates with poorer overall outcomes and progression-free survival in ovarian cancer [183]. It has been proposed that NLR can be considered a biomarker to help define ovarian cancer progression [184].

There is evidence that neutrophils can promote metastasis and immunosuppression in ovarian cancer. TANs release NETs in the omentum and thereby create a permissive niche that helps ovarian cancer cells seed and grow [185]. In addition, NETs can recruit and modulate other immune cells (for example, innate-like B cells producing IL-10), thereby promoting immune suppression and metastatic outgrowth [186,187,188]. Presence of G-CSF and MUC16 in the TME can promote this neutrophil phenotype [189,190]. Furthermore, a NET-related signature including genes such as ELN, FBN1, IL1B, LCN2, MMP2, MMP9, RAC2 and SELL was proposed for monitoring ovarian cancer prognosis prediction and therapy assessment [188].

Finally, it has been postulated that metabolic reprogramming, including glycolysis, fatty acid metabolism, and amino acid metabolism, helps TANs survive in the TME and supports protumor functions [191]. For example, glycolytic reprogramming in TANs promotes survival of these cells, which can then induce angiogenic and metastatic processes in ovarian cancer. Fatty acid metabolic reprogramming allows these cells to continue to produce ROS under glucose-deprived conditions, and amino acid metabolic reprogramming causes depletion of amino acids that can be used by immune cells, thereby inducing an immunosuppressive milieu [191].

## 19. Innate Lymphoid Cells (ILCs) in Ovarian Cancer

Innate lymphoid cells (ILCs) are a family of lymphoid cells with similar phenotypes and functions to T cells, in particular their cytokine response, but lack a T cell receptor and therefore are not subjected to clonal selection and expansion when stimulated. ILCs, being innate immune cells, react promptly to signals from infected or injured tissues and produce cytokines that mirror those of T cell subsets [192]. ILCs are classified into five different subsets: natural killer (NK) cells, ILC1s, ILC2s, ILC3s, and lymphoid tissue inducer (LTi) cells. While NK cells are found systemically and are involved mainly in cytotoxic activities, the other subsets are confined to tissues where they participate in local specific immunity [193]. The main function of NK cells and ILC1s is the elimination of tumor cells due to their cytotoxic (NK) and inflammatory cytokine production (ILC1) capabilities. On the other hand, ILC2 and ILC3 produce cytokines that might promote or control tumor development depending on the microenvironment [193].

In ovarian cancer, NK cells are frequently suppressed. NK cells recovered from tumor sites and malignant ascites usually show reduced cytotoxic activity compared with healthy blood NK cells [194]. Malignant ascites factors (high TGF-β, altered electrolytes, lipids) actively inhibit NK cell function [194,195]. In addition, HGSOC spreads across peritoneal surfaces where tissue-resident ILCs and NK cells can interact with tumor cells and ascites factors; this localization shapes their phenotype and function [196]. Tumor factors such as TGF-β can modulate ILC activity to convert them into cells that support tumor growth by producing factors that promote angiogenesis, tissue remodeling or immune suppression [197]. ILCs have been identified in ascites from HGSOC patients by means of multicolor flow cytometry using both surface and intracellular markers, which showed up to 17 different ILC clusters, highlighting the complexity of the immune regulation occurring in the TME [198].

## 20. B Cells in Ovarian Cancer

B cells can be found both in ascites and in solid tumors in ovarian cancer. It is a matter of current debate whether B cells help promote or suppress tumor growth, and it probably depends on the particular characteristics of each patient. For example, in ascites it has been shown that B cells can present an immunosuppressive phenotype, characterized by production of IL-10. These regulatory B cells (or Bregs) might be able to promote Tregs and suppress immune responses [117]. On the other hand, the presence of B cell infiltrates, in particular those associated with tertiary lymphoid structures, is associated with better patient prognosis [199]. Furthermore, intratumoral B cells in ovarian cancer patients produce antibodies, particularly of the IgA isotype, that target tumor antigens and are associated with better prognosis [118]. These IgA antibodies redirect myeloid cells against extracellular oncogenic drivers, causing tumor cell death in a process mediated by IgA transcytosis through the epithelial ovarian cancer cells [118].

## 21. T Cells in Ovarian Cancer

Despite ovarian cancer being considered a “cold tumor” due to its low TMB and consequent low number of neoantigens, a foundational study for ovarian cancer immunotherapy described an association between tumor-infiltrating lymphocytes (TILs) in HGSOC solid tumors and survival [200]. Further studies confirmed this association, describing a positive correlation between the amount of CD8^+^ TILs or the CD8/Treg ratio and favorable clinical outcomes [201,202,203]. Indeed, a large-scale analysis showed that CD8^+^ TILs vary by histotype, and that HGSOC tumors show the highest association between CD8 TIL infiltration and survival regardless of the extent of residual disease or first-line chemotherapy treatment [203]. Interestingly, a study focused on long-term ovarian cancer survivors showed higher levels of combined intraepithelial CD8^+^ T cells and intra-stromal B cell levels compared with short-term survivors [204].

**Figure 3:**
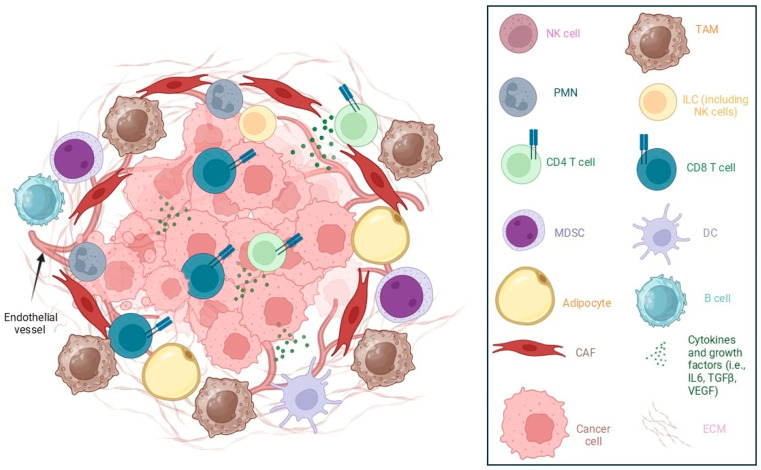
Ovarian cancer microenvironment (solid tumor). The figure presents the components of high-grade serous ovarian cancer solid tumors. CAF: cancer-associated fibroblast, DC: dendritic cell, IL6: interleukin-6, ECM: extracellular matrix, ILC: innate lymphoid cell, MDSC: myeloid-derived suppressor cell, NK: natural killer, PMN: polymorphonuclear, TAM: tumor-associated macrophage, TGF: transforming growth factor, VEGF: vascular endothelial growth factor. Created with BioRender.com.

Solid tumors are infiltrated by both CD8 and CD4 T cells, but in many cases, markers of exhaustion such as TIM-3 and PD-1 are observed in those cells [205]. CD8 (albeit often dysfunctional) and CD4 T cells, including a high proportion of regulatory CD4 T cells (Treg), are also present in ovarian cancer malignant ascites [206]. Figure 3 summarizes the main components of the ovarian cancer solid tumor microenvironment.

The presence of infiltrating T cells and, in particular, the association of high CD8 and low Treg infiltration with better outcomes opened the door for ovarian cancer immunotherapies, including dendritic cell vaccinations or immune checkpoint inhibitor therapies aiming to induce or reactivate the patients’ immune response against the tumors. These strategies have been investigated in many preclinical studies and clinical trials [207,208]. In recent years, the use of immune checkpoint inhibitors (ICI) for cancer therapy has successfully increased overall survival in patients with melanoma, bladder cancer and some types of lung cancer. The strategy aims to activate T cells that are typically exhausted due to tumor factors. Unfortunately, ICI therapy has not been very successful in ovarian cancer, possibly due to the highly immunosuppressive tumor microenvironment; therefore, alternative therapeutic strategies are needed [207].

## 22. Effect of Metformin on Tumor Microenvironment Components

Metformin can affect the activity of immune cells. In vitro and in vivo studies have found that metformin can affect the NLRP3 inflammasome signaling cascade, effectively reducing inflammation. In one study, it was observed that metformin decreased cellular levels of NLRP3 in LPS-induced lung endothelial cells [209,210]. These results were replicated in many different cell types, including neurons and kidney cells, as well as immune cells such as macrophages [211,212,213,214]. Notably, in combination with resveratrol, metformin prevented the formation of reactive oxygen species in the adipose tissue of T2DM mouse models [215]. It is thought that NLRP3 activity is restricted due to the activation of the AMPK pathway triggered by metformin [216,217].

Metformin has been found to rescue CD8^+^ tumor-infiltrating lymphocytes from hypoxia-induced immunosuppression, apoptosis and exhaustion in several cancer models, including melanoma, lung cancer, leukemia, breast cancer, renal cancer and lymphoma, among others [218,219]. Programmed death ligand 1 (PD-L1) checkpoint inhibitors are a different type of anticancer immunotherapy. PD-L1 is often expressed by cells in the tumor microenvironment, such as tumor-associated macrophages, and contributes to tumor progression by binding to and decreasing the activity of T cells, ultimately helping cancer cells go undetected by the immune system. In a mouse model of ovarian cancer, it has been found that metformin significantly reduced tumor growth, increased CD8^+^ T cell infiltration, and improved combinatorial therapies with anti-PD-L1 antibodies [220]. Furthermore, in a model of ovarian cancer, it was shown that metformin, as a single agent, promoted CD8^+^ T cell and NK infiltration, inducing a robust antitumor response [221]. On the other hand, the literature search was unable to identify any report on the action of metformin on CD4 T cells in the context of ovarian cancer, which could be a promising topic for future studies, considering the central role that these cells play in the ovarian cancer microenvironment.

Another way that metformin can affect the TME is by inhibiting MDSC recruitment. MDSC accumulation in tumors is known to contribute to immunosuppression. In a study conducted on metformin’s effect on MDSCs in ovarian cancer, researchers found that the enzymatic activity of CD39^+^ and CD37^+^ MDSCs decreased in the presence of metformin, thus downregulating the immunosuppressive effect of MDSCs [222]. In the TME, CD39 and CD73 act together to convert pro-inflammatory extracellular ATP into immunosuppressive adenosine.

Additionally, it has been reported that metformin can promote antitumor immune responses in the TME by inducing immunogenic cell death (ICD) of ovarian cancer cells. This mechanism can provide DCs with appropriate tumor antigens to activate T cell responses. A2780 and SKOV3 cells treated with metformin showed characteristics of ICD (i.e., expression of calreticulin on the cell surface and release of ATP and HMGB1 protein), thus generating antigenic tumor material that was able to activate dendritic cells in vitro [78].

In ovarian cancer, metformin also has the capacity to modulate fibroblast biology. CAFs have the ability to promote tumor growth, for example by producing cytokines and growth factors that help tumor cell growth and survival. It was found that ovarian cancer patients treated with metformin and cisplatin harbored CAFs with lower levels of IL6 secretion than those treated with cisplatin alone and that metformin decreased the ability of fibroblasts to promote 3D organotypic coculture and murine xenograft ovarian cancer growth [223].

Although no information exists at present about the effect of metformin on neutrophils in ovarian cancer, in samples of patients with T2DM and colorectal cancer, metformin could modulate neutrophil biology by decreasing their capability to generate NETs [224]. It is tempting to speculate that this drug can induce a similar effect in ovarian cancer. Similarly, albeit no information exists on the effect of metformin on B cells in the context of ovarian cancer, metformin was able to improve B cell function in T2DM patients and decrease markers associated with a senescence-associated secretory phenotype in these cells [225].

Finally, metformin has been demonstrated to exert a different effect on lean or obese mice harboring ovarian cancer tumors, showing a higher effect on tumor growth in obese compared to lean mice [226]. Metabolic differences between tumors developed in obese versus lean mice are at the root of this response. In the model described in Han et al. (2017), tumors generated in obese mice had impaired mitochondrial complex II function, so inhibition of complex I by metformin led to a strong impairment of mitochondrial oxidative phosphorylation [226]. This determined that tumor cells only relied on glycolysis for generation of ATP [226]. Interestingly, metformin was able to inhibit adipogenesis and the ovarian tumor-promoting effects driven by adipocytes, for example proliferation of ID8 cells [227]. The inhibition of adipogenesis in these cells was mediated by AMPK activation.

**Figure 4:**
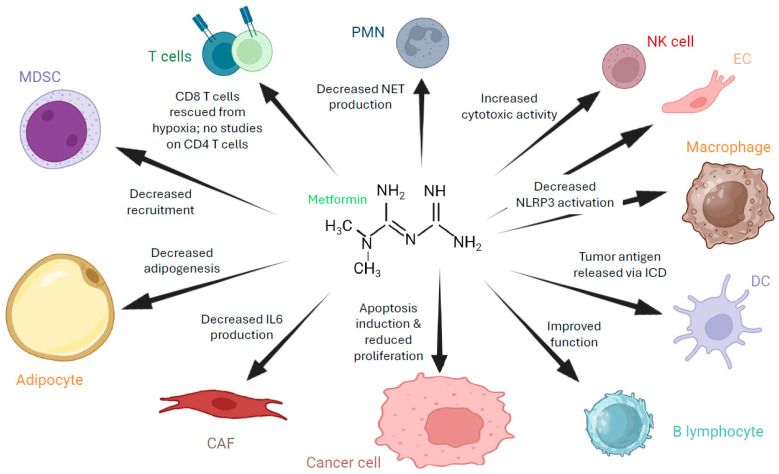
Main effects of metformin on different cell types present in the ovarian cancer TME. This figure summarizes the effect of metformin on cells present in the microenvironment of ovarian cancer. CAF: cancer-associated fibroblast, DC: dendritic cell, IL6: interleukin-6, ICD: immunogenic cell death, MDSC: myeloid-derived suppressor cell, NET: neutrophil extracellular trap, NK: natural killer, NLRP3: NLR family pyrin domain containing 3, PMN: polymorphonuclear. Created with BioRender.com.

A summary of metformin action on cancer cells and other cells from the TME is presented in Figure 4.

## 23. Clinical Trials with Metformin for Ovarian Cancer

Metformin has been evaluated in clinical trials for ovarian cancer in combination with first-line chemotherapy drugs (paclitaxel plus carboplatin). In a Phase I clinical trial, it was shown that metformin, given at 1000 mg three times a day, was safe with standard chemotherapy [228]. A Phase I study of sapanisertib (mTORC1/2 inhibitor) plus metformin in advanced solid tumors (including ovarian cancer) showed that the combination was generally tolerable, with early signs of antitumor activity [229]. Although in an open-label pilot trial with this combination it was reported that metformin did not significantly increase progression-free or disease-free survival, results from two Phase II studies with this combination indicated that the treatment appeared to modulate the IGF-1 axis by suppressing the increase in IGF-1 while preserving IGFBP-1 levels [228]. A Phase II (non-randomized) trial using this drug combination showed moderate efficacy and manageable tolerability, suggesting that metformin may enhance chemotherapy response [230].

Interestingly, a study of clinical databases to survey survival rate in ovarian cancer treated with metformin combined with chemotherapy showed significantly longer overall survival rates for patients treated with metformin as part of a neoadjuvant therapy than for patients treated without metformin [231].

Table 1 provides a description of several recent or ongoing clinical trials for ovarian cancer in which metformin was used in combination with established chemotherapeutic agents such as paclitaxel and carboplatin, PARP inhibitors and immune checkpoint inhibitors or experimental drugs such as LY3023414 (a PI3 kinase alpha inhibitor), among others. Of the seven clinical trials presented in Table 1, only one has posted results. This Phase II trial investigated the impact of metformin on cancer stem cell number and carcinoma-associated mesenchymal stem cells, together with clinical outcomes, in nondiabetic patients with advanced-stage epithelial ovarian cancer [121]. In this study, a significant reduction in the cancer stem cell population was observed, together with an alteration of DNA methylation of cancer-associated mesenchymal cells, which led to an increase in chemosensitivity. Finally, better-than-expected median overall survival was observed in metformin-treated stage II–III patients, supporting investigating metformin as an adjuvant drug in a Phase III clinical trial (NCT01579812) [96].

On the other hand, in a study in which data from three prospective, Phase III, randomized controlled trials (AGO-OVAR 11/ICON 7, AGO-OVAR 12, and AGO-OVAR 16) and one Phase II randomized controlled trial (AGO-OVAR 15) were pooled and analyzed, combinatorial treatment with metformin and statins had no significant impact on survival in patients with primary ovarian cancer [232].

As described in previous sections, metformin has an effect on immune cells present in the tumor microenvironment; however, it is noteworthy that most of the studies described in Table 1 focus on progression-free survival, but no immunological parameters were proposed to be assessed. Only NCT01579812 investigated further parameters such as the presence of cancer stem cells. Clinical trial NCT03378297 indicates that relevant biomarkers will be investigated, but those biomarkers are not described. Published data from ovarian cancer clinical trials from the researchers involved in clinical trial NCT03378297 indicate the use of serum cytokines (i.e., IL-4, IL-6, IL-7, CXCL10) or serum lipoproteins as biomarkers, but the studies do not include patients under metformin treatment [233,234]. It would be relevant to investigate immunometabolic parameters in future clinical trials using metformin as a combinatorial therapy for ovarian cancer.

Therefore, more clinical studies need to be performed to determine the relevance of metformin as a therapeutic agent in ovarian cancer. One relevant issue in order to consider the relevance of metformin as an anticancer agent for ovarian cancer is the possibility of deleterious effects. It is well known that metformin activates AMPK, which is considered one of the major redox hubs in mammalian cells together with nuclear factor erythroid 2 (NRF2), nuclear factor-κ light chain-enhancer of activated B cells (NF-κB), hypoxia-inducible factor (HIF), estrogen-related receptor (ERR), forkhead box O transcription factor (FOXO) and peroxisome proliferator-activated receptor-γ co-activator 1α, among others, as reviewed in detail by Sies et al. [235]. It has been shown that antioxidants can increase the development of metastasis in preclinical models of metastasis [236,237]. Considering that metformin can act as an indirect antioxidant by reducing oxidative stress, further studies using network pharmacology based on the application of omics data, bioinformatics, and machine learning tools should be performed to provide a comprehensive understanding of the effect of metformin on the metabolism of ovarian cancer cells and its role as a possible therapeutic agent for ovarian cancer [238].

## 24. Conclusions

Metformin, a drug used for T2DM treatment, has been shown to possess anticancer properties against ovarian cancer cells (mouse and human), effectively impairing tumor growth in preclinical models, and has been evaluated in clinical trials in combination with chemotherapeutic drugs. An interesting effect was observed on cancer stem cells in a Phase II clinical trial, inducing a reduction in these cells in treated patients. The effect of metformin on ovarian tumor cells might be multipronged. It can increase AMPK signaling, which in turn can decrease fatty acid oxidation and biosynthesis, as has been reported in other types of cancer cells. In addition, some studies suggest that it can impair protective autophagy, thereby decreasing overall cell viability. Activation of AMPK will increase glucose uptake by the tumor cells, which can modify the metabolic status of the cells. Indeed, it can be speculated that a metabolic profile of the individual patient’s tumor cells could indicate if they are a good match for metformin treatment. Importantly, since the metabolic status of tumor cells differs between ascites and solid tumors, these cells will be differentially targeted by metformin. Metformin can also target immune cells in the TME of ovarian cancer by decreasing immune suppression while activating T cells, which might improve immunotherapeutic responses.

## Figures and Tables

**Table 1:** Metformin in ovarian cancer clinical trials. This table summarizes data on clinical trials for ovarian cancer in which metformin was used as an anti-tumor agent. Data were collected from ClinicalTrials.gov.

Intervention	Official Study Title	NCT Number	Sample Size	Patient Characteristics	Outcome Measures	Key Findings
Metformin, Paclitaxel, and Carboplatin	A Phase II, Open-Label, Non-Randomized, Pilot Study of Paclitaxel, Carboplatin and Oral Metformin for Patients Newly Diagnosed With Stage II–IV Epithelial Ovarian, Fallopian Tube or Primary Peritoneal Carcinoma	NCT02437812	30	Patients with advanced-stage ovarian carcinoma treated with paclitaxel, carboplatin and metformin, but not under current metformin treatment	Measurement of progression-free survival	Not posted
Metformin, Carboplatin and Paclitaxel	Phase Ib Study of Metformin in Combination With Carboplatin/Paclitaxel Chemotherapy in Patients With Advanced Ovarian Cancer	NCT02312661	15	Patients with advanced-stage epithelial ovarian carcinoma	Determination of the recommended dose for metformin plus carboplatin and paclitaxel in a Phase II trial	Not posted
Zimberelimab and Metformin Hydrochloride	Zimberelimab Combined With Metformin in the Treatment of Recurrent Ovarian Clear Cell Carcinoma: A Pilot Study	NCT05759312	20	Patients with recurrent ovarian clear cell carcinoma	Assessment of complete and partial response in accordance with the RECIST 1.1 criteria	Not posted
Metformin, Acetylsalicylic acid, Olaparib and Letrozole	IMPACT: A Phase 0 Randomized Window-of-Opportunity Study of Novel and Repurposed Therapeutic Agents in Women Triaged to Primary Surgery for Advanced Epithelial Ovarian Cancer in Stages IIIa–IV	NCT03378297	26	Patients with a diagnosis of advanced ovarian, tubal or primary peritoneal cancer	Changes in the expression of defined biomarkers as determined by histology	Not posted
Metformin	A Phase II Evaluation of Metformin, Targeting Cancer Stem Cells for the Prevention of Relapse in Patients With Stage IIC/III/IV Ovarian, Fallopian Tube, and Primary Peritoneal Cancer	NCT01579812	90	Patients with potential diagnosis of ovarian, fallopian, or primary peritoneal cancer	Percentage of patients alive without recurrence at 18 months	Tumors treated with metformin had a decrease in cancer stem cells and increased sensitivity to cisplatin
Letrozole; Abemaciclib; LY3023414; Metformin; Zotatifin; Gedatolisib	RESOLVE: letRozole abEmaciclib combinationS in endOmetriaL and oVarian cancEr: A Multi-Cohort Phase 2 Study of Letrozole/Abemaciclib Alone and in Combination With Metformin, Zotatifin and Gedatolisib	NCT03675893	180	Patients with histologically confirmed diagnosis of low-grade serous ovarian carcinoma	Progression-free survival	Not posted
Metformin hydrochloride and standard chemotherapy	A Randomized Placebo Controlled Phase II Trial of Metformin in Conjunction With Chemotherapy Followed by Metformin Maintenance Therapy in Advanced Stage Ovarian, Fallopian Tube and Primary Peritoneal Cancer	NCT02122185	110	Diagnosis of ovarian carcinomatosis	Progression-free survival	Not posted

## Data Availability

No new data were created for this manuscript.
